# Hybrid CNN–transformer model with BM3D and YOLOv8 for early detection of lung cancer in low-dose CT scans

**DOI:** 10.1038/s41598-026-43517-5

**Published:** 2026-03-15

**Authors:** Gagan Thakral, Umesh Kumar, Sapna Gambhir

**Affiliations:** 1 J. C. Bose University of Science and Technology, YMCA, Faridabad, India; 2 Krishna Institute of Engineering &Technology (KIET), Delhi-NCR, Uttar Pradesh Ghaziabad, India; 3 George Mason University, Fairfax, VA USA

**Keywords:** Lung cancer, Early detection, Low-dose CT scan, Deep learning, CNN, Hybrid model transformer encoder, Cancer, Computational biology and bioinformatics, Engineering, Health care, Mathematics and computing, Medical research, Oncology

## Abstract

Lung cancer remains the primary cause of cancer-related deaths throughout the world. The main reason behind this is late diagnosis and the restrictions in the manual interpretation of imaging data. In these days Low-Dose Computed Tomography (LDCT) has been widely adopted for early screening. LDCT contains Low Dose x-rays as compared to the normal CT scan. But the existence of noise and subtle nodular patterns often impairs diagnostic accuracy. In this study, authors proposed a novel hybrid deep learning model which uses BM3D for pre-processing and YOLOv8 for segmentation. Further this model integrates Convolutional Neural Networks (CNNs) with Transformer Encoders to enhance the early detection of lung cancer using LDCT scan images. The model powers the spatial feature extraction with the help of CNNs and the contextual reasoning capability of Transformers to achieve superior classification performance. In this work, during the training of model BM3D filtering (advanced image preprocessing technique) are applied to reduce noise and enhance structural details. Further YOLOv8 is used for segmentation. The proposed hybrid model achieved 93.8% sensitivity, 95.1% accuracy, 94.4% F1-Score, 96.2% Specificity, 0.92 Dice Metric and 0.97 AUC for classification. Experimental results demonstrate that the proposed model outperforms existing models in terms of accuracy, precision, recall, AUC, and Dice coefficient. These findings suggest that the hybrid model holds strong potential as a robust tool for early lung cancer screening and clinical decision support.

## Introduction

The World Health Organization (WHO) reports that lung cancer remains one of the most dominant and deadly forms of cancer. The organization accounts for approximately 1.8 million deaths annually^1^. The late analysis of the disease is primarily attributed to the high death rate. This will also suggestively limit the usefulness of available treatment options^2^. Early detection of lung cancer is very important to improve patient’s diagnosis and existence rates. Timely detection can lead to more successful and beneficial outcomes.

In these days, LDCT becomes as emerging and widely accepted non-offensive imaging modality for lung cancer screening^3^. With the help of LDCT Pulmonary nodules are detected at an early stage with reduced radiation experience. Sometimes small cases may be misdiagnosed due to noisy and complex images produced by LDCT scans, especially when they are small or have irregular boundaries. However, LDCT scans often produce misinterpretation and false positives. The need for accurate, robust, and automated diagnostic tools is increasingly vital in supporting radiologists and enhancing clinical decision-making.

Significant potential in medical image investigation has been established by recent advancements in deep learning, mainly for tasks such as detection, segmentation, and classification. CNNs have shown amazing performance in obtaining spatial features from medical images^4^. Natural Language Processing has been effectively utilized to learn long-range relationships and global information in image data. While each of these models has its own merits, models using CNNs alone or Transformers alone might be lacking in utilizing both local and global features of an image effectively^5^.

In order to overcome these limitations, this research advocates the development of a new hybrid deep learning model that employs CNN and Transformer Encoder architectures for the early detection of lung cancer from LDCT scan images. The new model synergizes the local feature extraction ability of CNNs and the contextual reasoning capability of Transformers and results in a holistic, highly accurate classification system. Additionally, advanced preprocessing techniques, like BM3D filtering, are used to improve image quality prior to model training.

This paper aims to demonstrate that the proposed hybrid model significantly improves detection sensitivity, specificity, and accuracy compared to traditional deep learning models. The proposed model is validated over the publicly available LIDC-IDRI datasets. The obtained results are benchmarked over the several state-of-the-art frameworks.

“Related work” is used to describe the related work and research gap. The proposed methodology, pre-processing, dataset description, proposed methodology, training, and optimization are described in “Methodology”. Experimental results, evaluation metrics, comparative analysis, qualitative results, discussion and challenges are described in “Experimental results and discussion”. Finally, “Conclusion and future work” represents the conclusion and future scope of the study.

## Related work

Early diagnosis of lung cancer has been a major focus of medical imaging research, as the disease is highly fatal and often detected at an advanced stage^6^. LDCT has proven to be an accepted screening tool, providing a trade-off between sensitivity and less radiation exposure. Nevertheless, LDCT scan interpretation is generally hindered by image noise, anatomical complexity, and heterogeneity of nodule presentation, thus necessitating the use of automated approaches.

### Traditional machine learning approaches

Early attempts at computer-aided diagnosis (CAD) of lung cancer included classical machine learning methods based on hand-crafted features. Techniques containing random forests, support vector machines (SVM) and k-nearest neighbors (k-NN) were employed in aggregation with features such as shape, texture and intensity^7^. While these methods generated encouraging results, they were incapable of generalizing over large and diverse datasets because of the limited expressiveness of manually crafted features.

### Deep learning-based models

Over the past few years, deep learning models, especially CNNs, have formed the backbone of lung nodule detection and classification tasks^8^. As an example,^9^ used a multi-view CNN structure to enhance false positive reduction in lung nodule detection. Likewise, Liao et al. proved that end-to-end learning from LDCT data can be done using deep CNNs effectively. While CNNs are best at learning local features, they are restricted in their ability to learn long-range dependencies and global context.

### Transformer-based approaches

Transformers, which were initially proposed for sequence modeling in NLP, proved successful in computer vision applications as well. Vision Transformers (ViTs) have been used in medical imaging to learn global contextual interactions. Dosovitskiy et al.^10^ proposed the ViT model, where images are split into patches and are processed by Transformer encoders. In medical imaging, the application of ViTs has been the focus of recent research in order to achieve better interpretability and performance, although standalone Transformer models tend to consume large amounts of data and computational power.

### Hybrid CNN-transformer models

Durgam et al.^11^ explain that early approaches trusted strongly on CNN models, which directed strong feature extraction capability. But these CNN models struggled with overfitting, generalization, and limited performance under varying imaging conditions. Authors also explain that several recent studies focused on ensemble learning, where multiple models are combined to improve diagnostic accuracy and reliability. But these models generally increase complexity and demand high computational resources. To handle these gaps, the reviewed literature supports hybrid solutions that combine segmentation + classification pipelines. Segmentation is used to find lung regions and classification is used to find cancerous or non-cancerous. This will increase the reliability of the system. The paper positions its proposed framework (CanNS) in this direction by integrating Swin-transformer segmentation and a deep learning classifier. To take advantage of both frameworks, hybrid models that integrate CNNs and Transformers have been explored. These models usually employ CNN layers for low-level feature extraction and Transformer encoders to model global context^12^. TransUNet was proposed by^13^ for medical image segmentation with better performance than CNNs alone. Few efforts have been made to use such hybrid models specifically for LDCT-based lung cancer detection.

### Research gap

Despite significant progress with time, most prevailing models collapse to effectively denoise LDCT scans. Sometimes these models ineffectively capture both local and global features. Multiple evaluation metrics, including accuracy, sensitivity, AUC, and dice coefficient, are often missing from comprehensive comparisons. The development of a hybrid CNN-Transformer framework integrated with advanced preprocessing techniques like BM3D is motivation for improved performance and robustness in early lung cancer detection.

## Methodology

### Dataset and preprocessing

LIDC-IDRI dataset^14^ is utilized for proposed model to detect Lung Cancer in early stages. This dataset contains LDCT scan images which are annotated by experienced radiologists. Images in this dataset are in DICOM format which contains a series of 2D slices that collectively form a three-dimensional (3D) volume of the lungs. This dataset contains 1,018 thoracic scans which are collected from 1,010 unique patients^15^. Table 1 is used to display some sample images from dataset. The scans contain more than 244,000 individual DICOM images which are available in the form of 2D slices. Each scan was annotated by four experienced radiologists which contains nodule characteristics like size, shape, and malignancy likelihood. Each scan also provides lung nodule locations. The dataset supports the creation and evaluation of automated computer-based detection and diagnosis systems by including labels that indicate the location and possibility of malignancy of discovered lung nodules in addition to imaging data. The labeling of the LIDC-IDRI dataset was performed using the malignancy likelihood score (1–5) assigned by radiologists. For each nodule, malignancy ratings were aggregated across available radiologists using the mean consensus score. Nodules with mean score 1–2 were labeled non-cancerous (benign). Nodules with mean score 4–5 were labeled cancerous (malignant). Nodules with a mean score of 3 were treated as ambiguous/indeterminate and were excluded to avoid uncertain ground truth. Labeling was performed at the nodule level, and all extracted slices/patches belonging to that nodule inherited the same label.

**Table 1:**
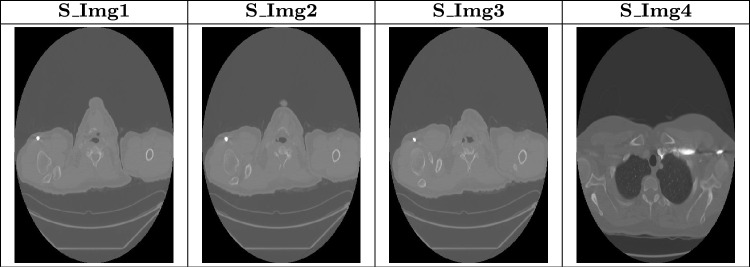
Sample images from LIDC-IDRI dataset.

Pre-processing of LDCT scan images is an initial step. It is very important step to develop accurate and reliable lung cancer detection systems. LDCT images contain low dose of X-rays as compared to the normal CT scan images. These images may suffer from variability in resolution, noise, and irrelevant anatomical structures. This may negatively affect the performance of Lung nodule detection system. The dataset is processed using the different preprocessing methods^16^. Whichever performs best based on SSIM value and PSNR is used.

Segmentation techniques also play an important role in the estimation of LDCT scan images for the detection of lung cancer. It is basically used for separating the lung area from neighboring anatomical structures such as muscles, bones, and fat tissues^17^. The focus of the segmentation is on the relevant areas where nodules are most likely to be present. Because accurate segmentation eliminates irrelevant data and reduces noise and it will improve the transparency and accuracy of subsequent processing steps. It also facilitates more effective nodule localization and feature extraction. Furthermore, segmentation establishes 3D reconstruction of lung nodules and volume-based analysis. It also provides more detailed understandings into their size, shape, and growth patterns. The absence of adequate segmentation algorithms may mistakenly identify normal tissues or artifacts as nodules which may increase the likelihood of false positives or missing diagnosis. Therefore, lung segmentation is essential for improving the sensitivity and specificity of lung cancer detection systems using LDCT images.

### Proposed hybrid model

**Fig. 1:**
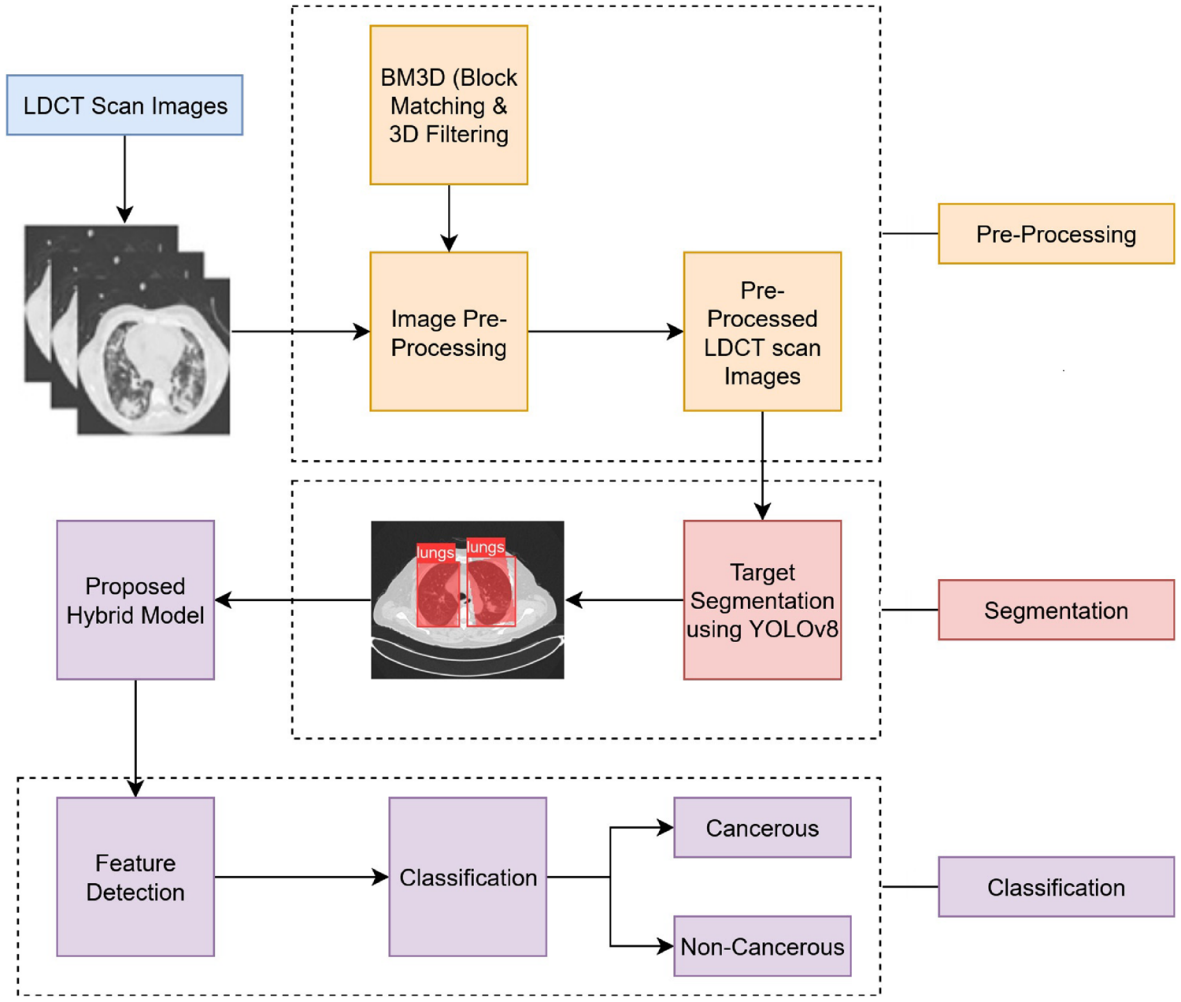
A novel hybrid CNN–transformer model with BM3D and YOLOv8 for early detection of lung cancer in low-dose CT scans.

A Novel Hybrid Deep Learning Model for Lung cancer detection from LDCT scan images is described in Fig 1. This figure initially displays some sample LDCT scan images. To Pre-process these LDCT images authors used various pre-processing techniques like Bi-Lateral Method, Gaussian Filter, Wavelet Method, TV Method, Invariant Wavelet, BM3D, and NLM Method^16^. Out of these techniques BM3D technique outperformed as compared to other techniques. The PSNR and SSIM (Quantitative evaluation) values are calculated for preprocessed images and authors logically found that BM3D is best suited to the selected dataset.

**Fig. 2:**
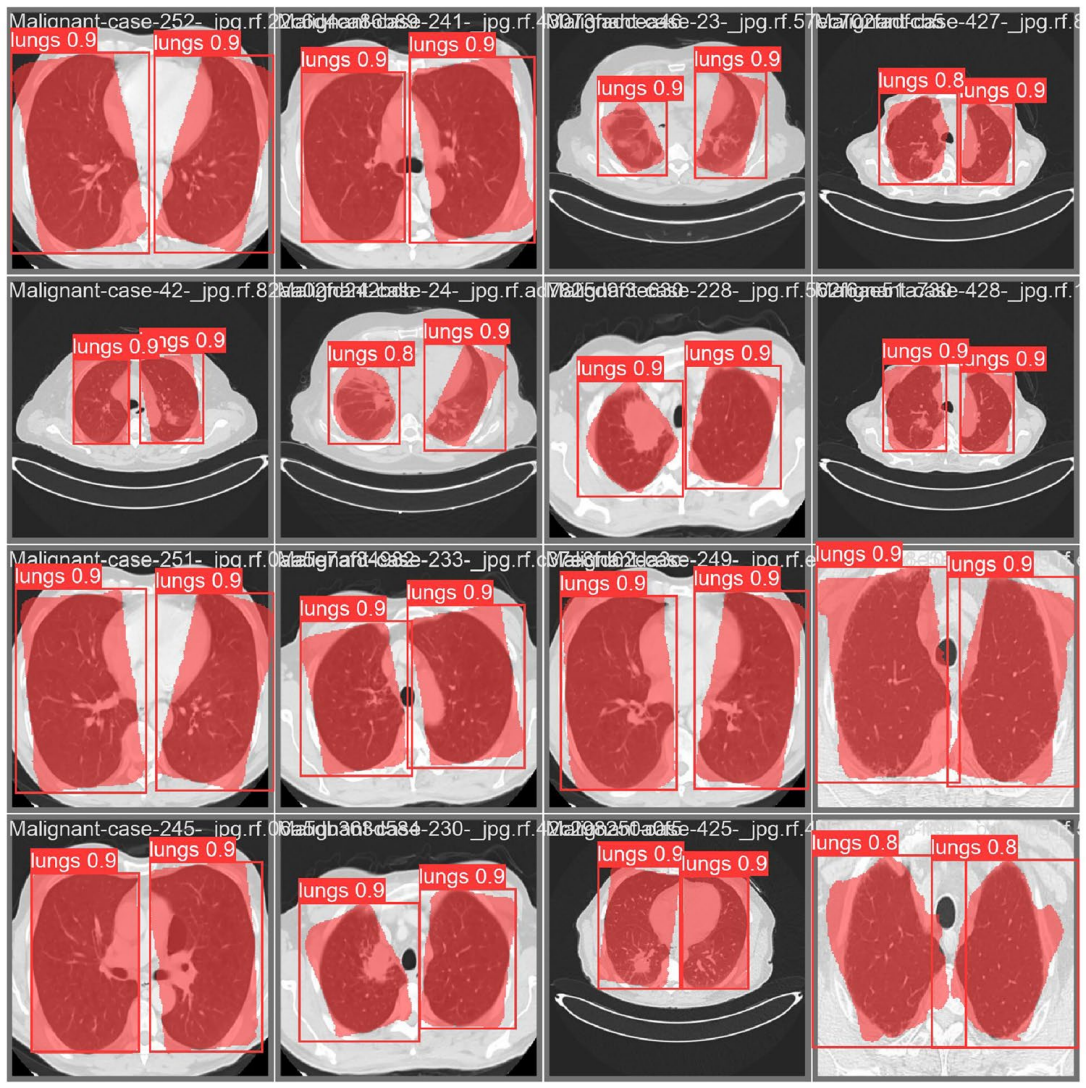
Qualitative results of the YOLOv8 lung segmentation, showing predicted lung regions with bounding boxes and confidence scores (0.8–0.9) across multiple test images.

Pre-processed LDCT scan images are then segmented into entire lung region (both lungs). The authors used Deep Learning based YOLOv8 Segmentation technique for this purpose^17^. This will remove non-lung background structures and provides a standardized lung ROI for subsequent analysis. The annotation format used is polygon-based segmentation labeling, which is compatible with YOLOv8 segmentation training. The annotations were created manually using the Roboflow annotation tool, where the lung boundaries were outlined using polygon masks. This tool was used to generate consistent segmentation labels for YOLOv8 training, testing and validation. The Fig. 2 is used to display the qualitative results of the YOLOv8 lung segmentation. This will show predicted lung regions with bounding boxes and confidence scores (0.8–0.9) across multiple test images^17^.

Further, for the feature detection from segmented images authors proposed a hybrid model. The proposed hybrid model combines the feature extraction power of CNNs with the contextual understanding capabilities of Transformer Encoders. The main aim of the proposed model is to improve accuracy in lung cancer detection system. The proposed model initially takes a LDCT scan image as input, following preprocessing and segmentation. This step ensures that the input to the proposed hybrid deep learning model is clean, consistent, and highlights the important lung regions for analysis. Resizing to standard dimensions and normalization are already applied in initial stages. Pre-processed images are fed into a CNN Model to learn low-level to mid-level features like edges, textures, and nodule shapes. The CNN layers efficiently capture the local spatial patterns essential for detecting suspicious regions or nodules. The CNN analyzes image data in layers that include convolution, activation, and pooling.

The fundamental mathematical formulations are shown below: The 2D convolution for an input image $I$ and a filter $F$ is given by:

1 $$\begin{aligned} S(p, q) = (I * F)(p, q) = \sum _{i=0}^{X-1} \sum _{j=0}^{Y-1} I(p + i, q + j) \cdot F(i, j) \end{aligned}$$

where $I(p, q)$ is the input pixel at position $(p, q)$, $F(i, j)$ is the filter value at position $(i, j)$, $S(p, q)$ is the resulting feature map at position $(p, q)$, $X \times Y$ is the size of the kernel.

### Activation function

ReLU a non-linear activation function is used to convolved the output:

2 $$\begin{aligned} A(p, q) = \max (0, S(p, q)) \end{aligned}$$

This non-linear function helps in learning complex patterns and provides non-linearity.

### Output size calculation using stride and padding

stride $\backslash$( b $\backslash$) and Padding $\backslash$( a $\backslash$) are used to maintain spatial dimensions. It also used to control output size:

3 $$\begin{aligned} \text {Output size} = \left\lfloor \frac{(W - F + 2a)}{b} \right\rfloor + 1 \end{aligned}$$

where $a$ denotes padding size, $b$ represents stride value, $W$ is height or input width, filter size is represented by $F$.

### Pooling operation

Max pooling layer is used to maintain the most crucial information while reducing the input feature maps such as width and height:

4 $$\begin{aligned} P(p, q) = \max _{(i,j) \in \text {pool region}} A(p + i, q + j) \end{aligned}$$

### Fully connected layer and flattening

The final feature map is flattened into a 1D vector $v$, then passed to a fully connected layer:

5 $$\begin{aligned} & v = \text {Flatten}(P) \end{aligned}$$

6 $$\begin{aligned} & y = W v + B \end{aligned}$$

where $v$ is input vector, $y$ is output vector, weight matrix is represented by $W$, bias is represented by $B$.

**Fig. 3:**
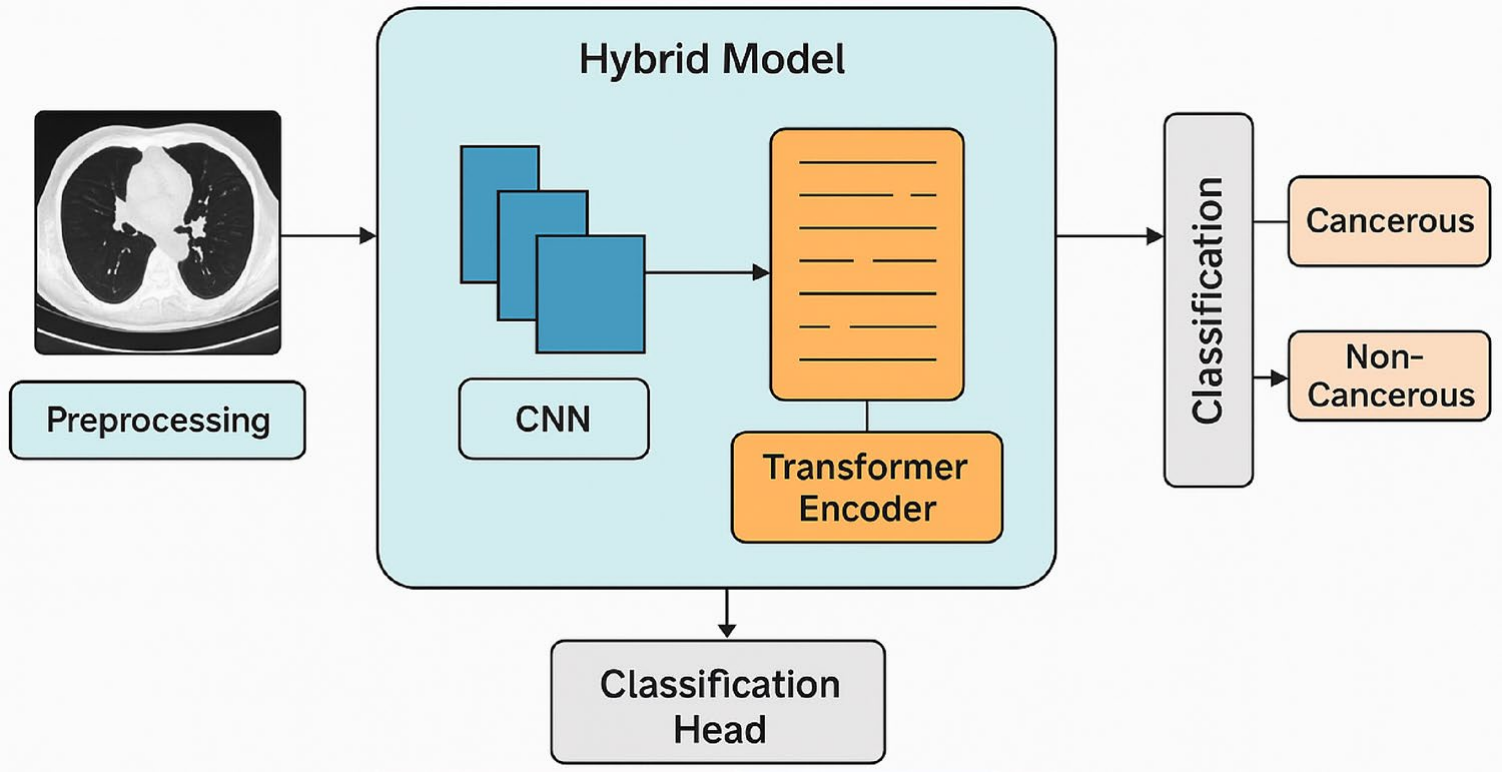
Proposed hybrid deep learning model for lung cancer detection from LDCT scan images.

**Fig. 4:**
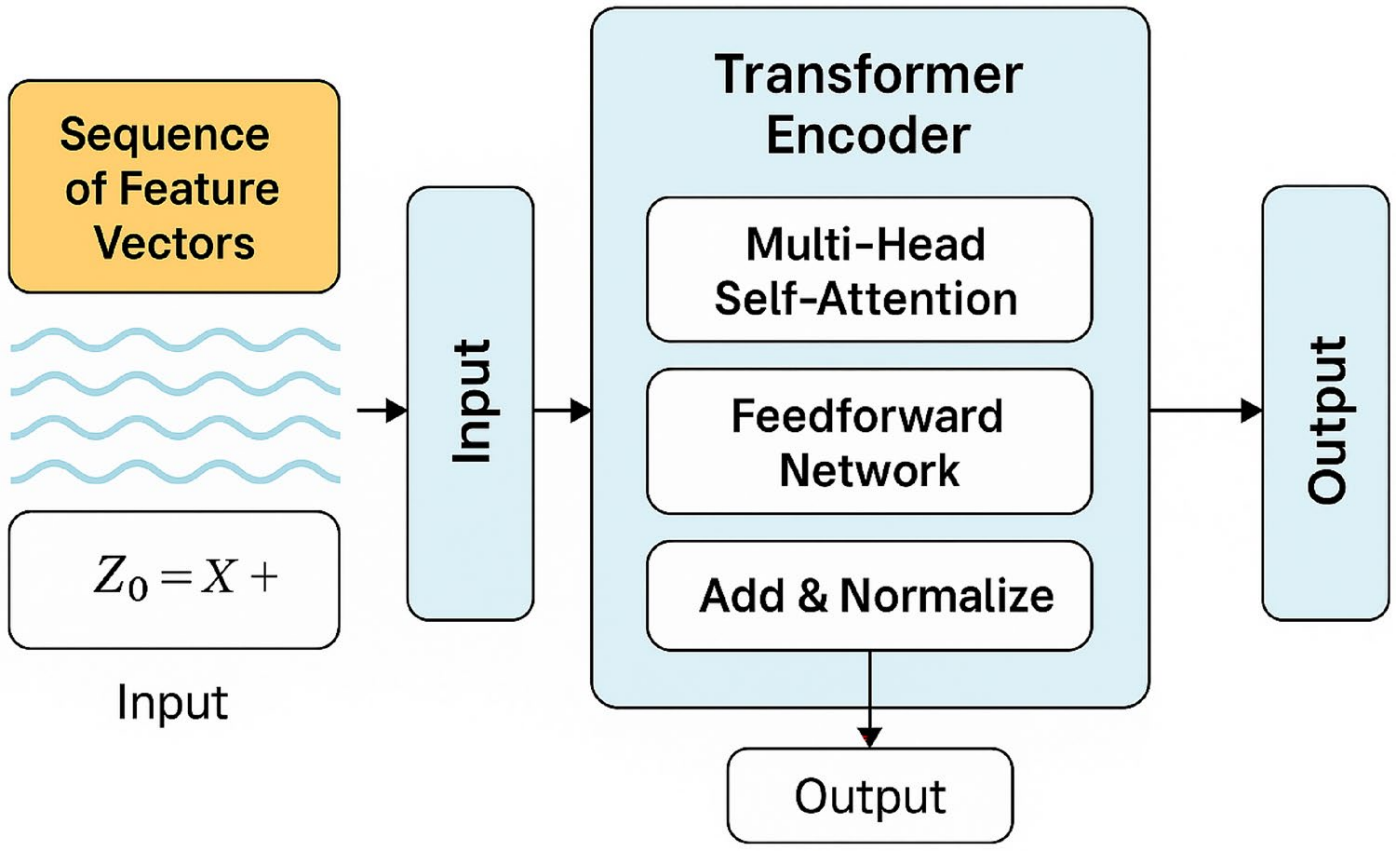
Detailed representation of transformer encoder.

These features produced by CNN are then fed to a transformer encoder. Figure 3 is used to describe hybrid model for classification. Figure 4 provides a detailed representation of transformer encoder. Proposed model Transformers, initially developed for NLP, are increasingly being applied in medical imaging to learn long-range relationships and global contextual dependencies among the spatial features of the lung image. This assists in recognizing complex patterns and correlations that CNNs might fail to capture, such as very subtle alterations to a larger extent. In this framework, the feature maps produced by CNN are flattened and inputted into the Transformer Encoder.

The transformer encoder’s input representation is as follows:

Assume that the CNN output is a feature map of shape $W \times H \times C$, where:

- $W$ represents the Width of the feature map,
- Height of the feature map is represented by $H$,
- $C$ represents the Number of feature channels.

$N = W \times H$ patches are created by Flatten the spatial dimensions:

7 $$\begin{aligned} X = [x_1, x_2, \ldots , x_N] \in \mathbb {R}^{d \times N} \end{aligned}$$

Every token $x_i$ is added using positional encoding and linearly projected:

8 $$\begin{aligned} Z_0 = E_{pos}+X \end{aligned}$$

### Multi-head self-attention (MHSA)

For each encoder layer compute the key, value, and query matrices:

9 $$\begin{aligned} K = ZW^K, \quad V = ZW^V,\quad Q = ZW^Q \end{aligned}$$

where $K, V, Q \in \mathbb {R}^{N \times d_k}$, learnable projection weights are represented by $W^K, W^V, W^Q$.

The scaled dot-product attention is calculated as:

10 $$\begin{aligned} \text {Attention}( K, V, Q) = \text {softmax}\left( \frac{K^TQ}{\sqrt{d_k}} \right) V \end{aligned}$$

The outputs are combined and projected for $h$ attention heads:

11 $$\begin{aligned} \text {MHSA}(Z) = W^O[\text {head}_1; \ldots ; \text {head}_h] \end{aligned}$$

### Feedforward neural network (FFN)

After that, each attention output is passed to a feedforward network:

12 $$\begin{aligned} \text {FFN}(z) = \max (0, zW_1 + b_1)W_2 + b_2 \end{aligned}$$

### Add & norm (residual connections)

Layer normalization and residual connections are applied:

13 $$\begin{aligned} & Z' = \text {LayerNorm}(Z + \text {MHSA}(Z)) \end{aligned}$$

14 $$\begin{aligned} & Z'' = \text {LayerNorm}(Z' + \text {FFN}(Z')) \end{aligned}$$

The outputs $Z''$ which is generated by the final encoder layer is passed to the classification head for lung cancer classification.

After that the combined feature map (CNN + Transformer) is passed with the help of a fully connected layer (classification head). This will generate a probability score, based on this score, model will predict images as malignant or benign.

### Classification head

The output which is represented by $Z''$ is generated by the final Transformer encoder layer. A rich representation of the input image patches is captured, both locally and globally. The final prediction is generated by passing this output to the classification head.

### Flattening and pooling

Global representations are created by applying operations such as flattening or global average pooling to the sequence of embedded tokens:

15 $$\begin{aligned} z_{cls} = \text {GlobalAvgPool}(Z'') \in \mathbb {R}^{d} \end{aligned}$$

In some architectures, the embedding of the [CLS] token is directly extracted from $Z''$.

### Fully connected layer

One or more fully connected layers are passed to the pooled or flattened feature vector:

16 $$\begin{aligned} y = \sigma (W z_{cls} + b) \end{aligned}$$

where $W \in \mathbb {R}^{C \times d}$ is weight matrix, $b \in \mathbb {R}^{C}$ is bias vector, $\sigma$ is activation function, (typically softmax for multi-class or sigmoid for binary classification), $y \in \mathbb {R}^{C}$ is output probability vector, $C$ is number of output classes ($C = 2$ for lung cancer detection (cancerous or non-cancerous)).

### Final prediction

The highest probability index is used to select the final predicted class:

17 $$\begin{aligned} \hat{c} = \arg \max _{i} y_i \end{aligned}$$

The presence of lung cancer is indicated by the class label $\hat{c}$.

### Training and optimization

Training and optimization play a critical role in fine-tuning the hybrid deep learning model to accurately classify LDCT scan images into cancerous and non-cancerous categories. To avoid data leakage, the LIDC-IDRI dataset was split at the patient level. It also ensures that all slices and the extracted nodule patches belonging to the same patient were included in only one subset. A 70%, 15%, and 15% patient-wise split was used for training, validation, and testing, respectively. The experimental setup for implementing the suggested hybrid model is described in Table 6. The CNN backbone architecture of the suggested Hybrid CNN-Transformer Model is shown in Table 2. The transformer encoder parameters used for feature sequence learning are shown in Table 3. The suggested Hybrid classification model’s training configuration is shown in Table 4. The total number of Trainable Parameters that the model uses is shown in Table 5. These tables are helpful since they offer enough information about training settings to accurately replicate the suggested hybrid CNN + Transformer model.

**Table 2:** CNN backbone architecture used in the proposed hybrid CNN–Transformer model.

Block	Layers	Kernel / stride	Output channels	Output Size (224$\times$224 input)
Input	–	–	1 (grayscale)	224 $\times$ 224 $\times$ 1
Conv Block 1	Conv + BN + ReLU	3$\times$3 / 1	32	224 $\times$ 224 $\times$ 32
Pool 1	MaxPool	2$\times$2 / 2	–	112 $\times$ 112 $\times$ 32
Conv Block 2	Conv + BN + ReLU	3$\times$3 / 1	64	112 $\times$ 112 $\times$ 64
Pool 2	MaxPool	2$\times$2 / 2	–	56 $\times$ 56 $\times$ 64
Conv Block 3	Conv + BN + ReLU	3$\times$3 / 1	128	56 $\times$ 56 $\times$ 128
Pool 3	MaxPool	2$\times$2 / 2	–	28 $\times$ 28 $\times$ 128
Conv Block 4	Conv + BN + ReLU	3$\times$3 / 1	256	28 $\times$ 28 $\times$ 256
Pool 4	MaxPool	2$\times$2 / 2	–	14 $\times$ 14 $\times$ 256
Conv Block 5	Conv + BN + ReLU	3$\times$3 / 1	512	14 $\times$ 14 $\times$ 512

**Table 3:** Transformer encoder settings used for feature sequence learning.

Parameter	Value
Input tokens	Flattened CNN features
Tokenization	$14 \times 14$ feature map flattened into **196 tokens**
Token dimension before projection	512
Linear projection	$512 \rightarrow 256$
Transformer embedding size ($d_{model}$)	256
Number of encoder layers	4
Multi-head attention heads	8
Feed forward network (FFN) hidden size	1024
Dropout	0.1
Activation	GELU
Positional encoding	Learnable positional embeddings
Output representation	Global average pooling over tokens

**Table 4:** Training configuration of the proposed hybrid classification model.

Training parameter	Value
Input image type	LDCT lung ROI (after YOLOv8 segmentation)
Input size	$224 \times 224$
Batch size	16
Optimizer	Adam
Initial learning rate	0.0001
Learning rate scheduler	ReduceLROnPlateau
Scheduler factor	0.5
Scheduler patience	10 epochs
Number of epochs	50
Loss function	Binary cross entropy (BCE)
Early stopping	Enabled
Early stopping patience	10 epochs
Metric monitored	Validation loss
Weight initialization	He initialization
Data augmentation	Rotation ($\pm 15^{\circ }$), horizontal flip, zoom, shift, brightness
Train/val/test split	Patient-wise split (70/15/15)

**Table 5:** Total trainable parameters.

Model component	Parameters
CNN backbone	$\sim$3.2 M
Transformer encoder	$\sim$2.1 M
Classification head	$\sim$0.3 M
Total parameters	$\sim$**5.6 M**

Below Algorithm 1 provides the details of training and optimization of hybrid model for lung cancer detection using LDCT images.

**Algorithm 1:**
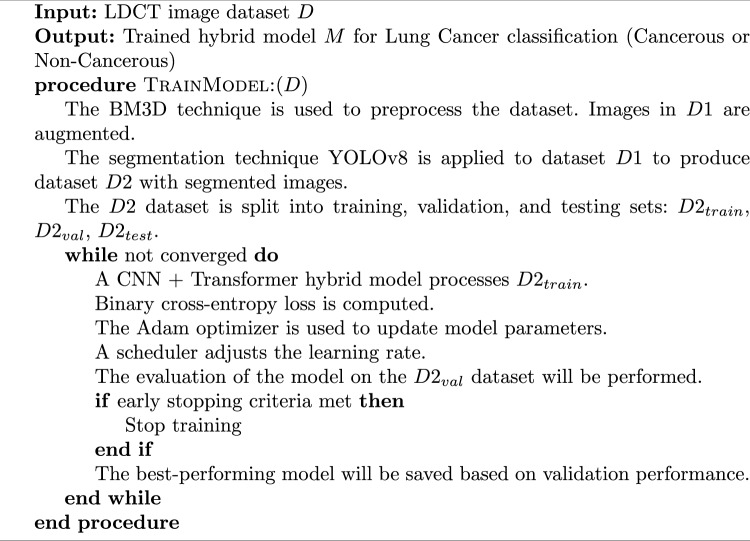
Training and optimization of hybrid model for lung cancer detection using LDCT images.

**Fig. 5:**
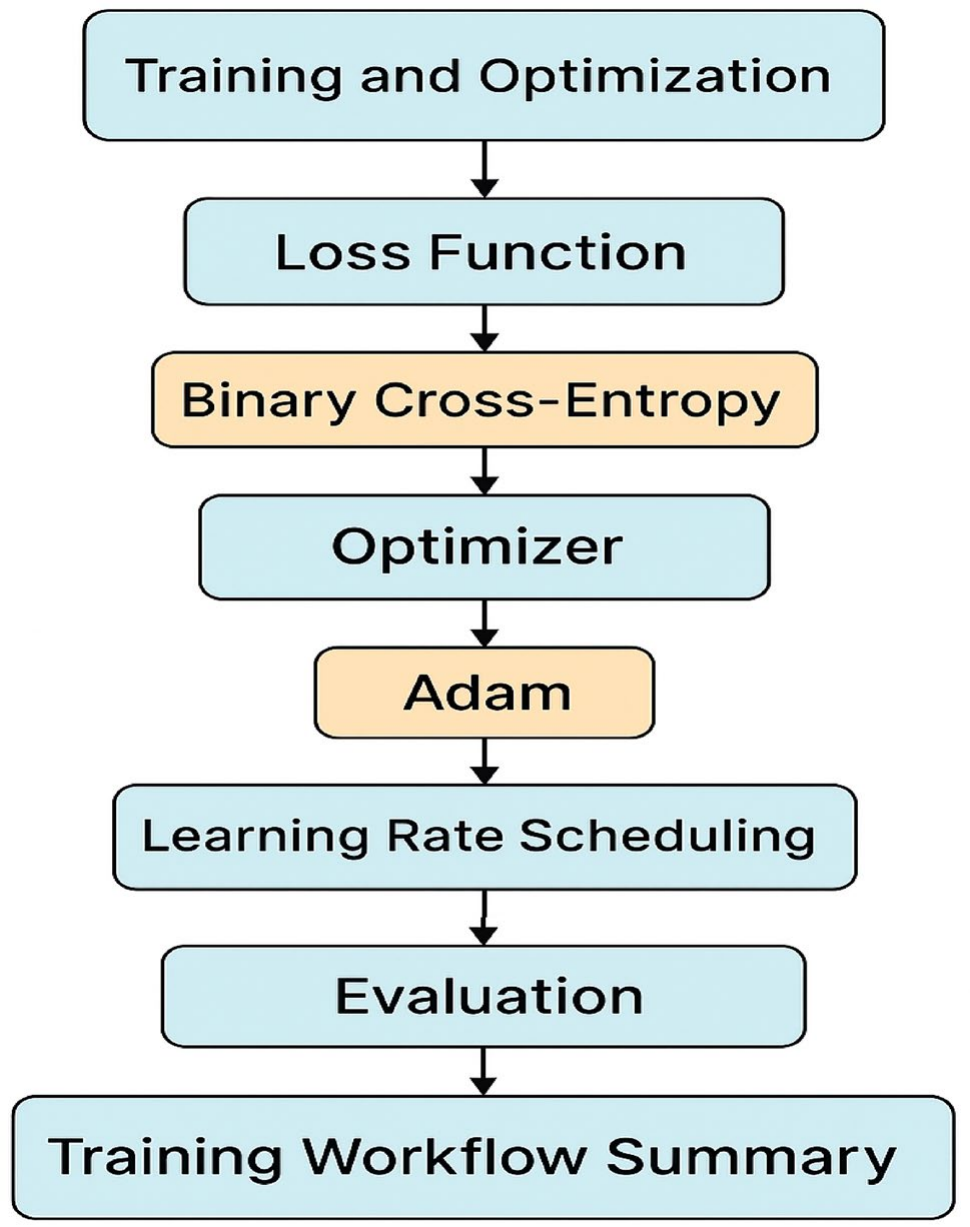
Training and optimization of hybrid model for lung cancer detection using LDCT images.

This process follows a supervised learning paradigm and involves loss calculation, backpropagation, parameter updates, and evaluation. The workflow also described in Fig. 5.

Loss function:

For binary classification, the Binary Cross-Entropy (BCE) loss is used to measure the discrepancy between predicted probabilities and actual labels:

18 $$\begin{aligned} \mathcal {L} = -\frac{1}{N} \sum _{i=1}^{N} \left[ y_i \log (\hat{y}_i) + (1 - y_i) \log (1 - \hat{y}_i) \right] \end{aligned}$$

where $y_i \in \{0,1\}$ is the true label for sample $i$, $\hat{y}_i \in (0,1)$ is the predicted probability, $N$ is the total number of samples.

Optimizer:

The Adam optimizer is used to adjust the weights of the CNN, classification head, and Transformer encoder. Adam combines the advantages of both AdaGrad and RMSProp:

19 $$\begin{aligned} \theta _{t+1} = \theta _t - \eta \cdot \frac{m_t}{\sqrt{v_t} + \epsilon } \end{aligned}$$

where $\theta _t$ is the parameter at step $t$, $m_t$ and $v_t$ are the first and second moment estimates, $\eta$ is the learning rate.

Learning rate scheduling:

A learning rate scheduler is used to dynamically adjust the learning rate during training. Techniques such as cosine annealing, step decay, or ReduceLROnPlateau help ensure convergence and prevent overshooting the minima.

Regularization:

To prevent overfitting and enhance generalization:

- Dropout is applied in the fully connected layers.
- L2 regularization (weight decay) penalizes large weights.
- Data augmentation includes transformations like rotation, scaling, and noise injection.

Early stopping was employed by monitoring the validation loss. Training was terminated if the validation loss did not improve by at least 0.0001 for 10 consecutive epochs, and the best-performing model was saved based on the minimum validation loss.

## Experimental results and discussion

### Evaluation metrics

- Accuracy: accuracy is a standard performance metric which is generally used to validate classification models^18^. It measures the proportion of correctly classified instances against the total number of instances classified. In simple words accuracy is the ratio of correctly classified samples to the total number of evaluated samples. It is a very simple and effective metric for the balanced dataset.
  20 $$\begin{aligned} \text {Accuracy} = \frac{TP + TN}{TP + TN + FP + FN} \end{aligned}$$
  where:
  - TP (true positives): when positive class (cancerous patients) is correctly identified as positive class (cancerous patients).
  - TN (true negatives): when negative class (non-cancerous patients) is correctly identified as negative class (non-cancerous patients).
  - FP (false positives): when negative class (non-cancerous patients) is incorrectly identified as positive class (cancerous patients).
  - FN (false negatives): when positive class (cancerous patients) is incorrectly identified as negative class (non-cancerous patients).
  A high accuracy specifies that the model performs well to differentiate between benign and malignant LDCT scan images. Generally, accuracy metric is considered for balanced dataset. However, it should be taken with attention for imbalanced datasets. For imbalanced datasets other metrics like recall, precision, and F1-score provide additional understandings.
- Sensitivity: Sensitivity is calculated as the percentage of actual positive cases (e.g., cancerous cases) that are correctly recognized by the model. It is the ratio of true positive to the sum of true positive and false negative. It is also known as True Positive Rate (TPR) or Recall^19^. In medical imaging, high sensitivity ensures that most patients with disease are detected correctly. In simple words it minimizes false negative or correctly identify positive cases (e.g., cancerous images).
  21 $$\begin{aligned} \text {Sensitivity} = \frac{TP}{TP + FN} \end{aligned}$$
  where:
  - $TP$: true positives—when positive class (cancerous patients) is correctly identified as positive class (cancerous patients).
  - $FN$: false negatives—when positive class (cancerous patients) is incorrectly identified as negative class (non-cancerous patients).
  A large value of sensitivity reflects the ability of the model to identify most of the genuine cancer cases.
- Specificity: Specificity is calculated as the percentage of actual negative cases (e.g., non-cancerous cases) that are correctly recognized by the model^20^. It is the ratio of true negative to the sum of true negative and false positive. It is also known as True Negative Rate (TNR). It helps assess the model’s ability to correctly identify negative cases.
  22 $$\begin{aligned} \text {Specificity} = \frac{TN}{TN + FP} \end{aligned}$$
  where:
  - $TN (true negatives)$: when negative class (non-cancerous patients) is correctly identified as negative class (non-cancerous patients).
  - $FP (false positives)$: when negative class (non-cancerous patients) is incorrectly identified as positive class (cancerous patients).
  A high specificity specifies that the model appropriately eliminates healthy people. This will reduce extra burden like medical care, lowering the needless worry, and expenses related to false positives.
- AUC: The Area Under the Receiver Operating Characteristic Curve (AUC-ROC) is a performance measurement metric. It is used to classify models at various threshold settings^21^. A higher value of AUC signifies better overall performance of the model. AUC mainly used to differentiate between positive and negative classes. ROC curve is a plot between the sensitivity (true positive rate) and specificity (false positive rate) at various threshold values. Generally, ROC curve is used to measure the performance of a binary classification model.
  23 $$\begin{aligned} \text {AUC} = \int _{0}^{1} \text {TPR}(FPR) \, dFPR \end{aligned}$$
  where:
  - $\text {TPR} = \frac{TP}{TP + FN}$ is the sensitivity (true positive rate),
  - $\text {FPR} = \frac{FP}{FP + TN}$ is the specificity (false positive rate).
  AUC values range from 0 to 1:
  - $$\text {AUC} = 1.0$$: perfect classifier,
  - $\text {AUC} = 0.5$: equivalent to random guessing( no discriminative power),
  - $\text {AUC} < 0.5$: inferior than random.
  A high AUC value in lung cancer detection means that the model can successfully distinguish between malignant and benign using LDCT scan images for all classification criteria.
- Dice Coefficient: The Dice Coefficient is a statistical measure which is used to measure the similarity between two samples^22^. It is developed by Sorensen-Dice, so it is also known as by Sorense-Dice index. It is commonly used to evaluate the performance of those models which work on image segmentation. Dice Coefficient is calculated by comparing the predicted segmentation with the ground truth mask. This metric is mainly used to measure the accuracy of the image segmentation models. It measures the overlap between the predicted region $P$ and the ground truth region $G$.
  24 $$\begin{aligned} \text {Dice} = \frac{2 |P \cap G|}{|P| + |G|} \end{aligned}$$
  The Dice score can also be represented in the form of true positives, false positives, and false negatives as:
  25 $$\begin{aligned} \text {Dice} = \frac{2TP}{2TP + FP + FN} \end{aligned}$$
  where:
  - $TP (true positives )$: when positive class (cancerous patients) is correctly identified as positive class (cancerous patients),
  - $FP (false positives )$: when negative class (non-cancerous patients) is incorrectly identified as positive class (cancerous patients),
  - $FN (false negatives )$: when positive class (cancerous patients) is incorrectly identified as negative class (non-cancerous patients)
  The Dice score ranges from 0 to 1:
  - $\text {Dice} = 1$: prediction and ground truth perfectly overlaps,
  - $$\text {Dice} = 0$$: no overlap.
  A higher Dice coefficient value always indicates better segmentation performance. Dice coefficient matrix is very important to estimate tumor segmentation or lung region in LDCT scan images.

### Comparative analysis

Table 7 shows the ablation study performed to better understand the impact of each module within our hybrid pipeline. Results without preprocessing with BM3D nor lung ROI segmentation with YOLOv8 on a CNN-only classifier (E1) shows baseline performance with an Accuracy of 89.4% and an AUC of 0.91. Next, adding preprocessing with BM3D (E2) improves classification performance with an Accuracy of 91.0% and an AUC of 0.93, showing that removing noise helps with LDCT scans classification. Adding segmentation of lung ROI with YOLOv8 (E3) also helps improving performance with an Accuracy of 92.2% and an AUC of 0.94, showing that zeroing in on the lungs during classification produces more reliable results. Combining both denoising with BM3D and lung ROI segmentation with YOLOv8 on top of a CNN classifier (E4) improves upon the previous models with an Accuracy of 93.1% and an AUC of 0.95. Switching out the CNN classifier for a Transformer-only classifier (E5) sees marginal gains with an Accuracy of 93.7% and an AUC of 0.96. This shows that learning global features produces a better classifier when all else is equal. Our full hybrid model with preprocessing using BM3D, lung ROI segmentation with YOLOv8, and a hybrid CNN + Transformer classifier (E6) outperforms all other experiments with an Accuracy of 95.1%, Sensitivity of 93.8%, and an AUC of 0.97.

**Fig. 6:**
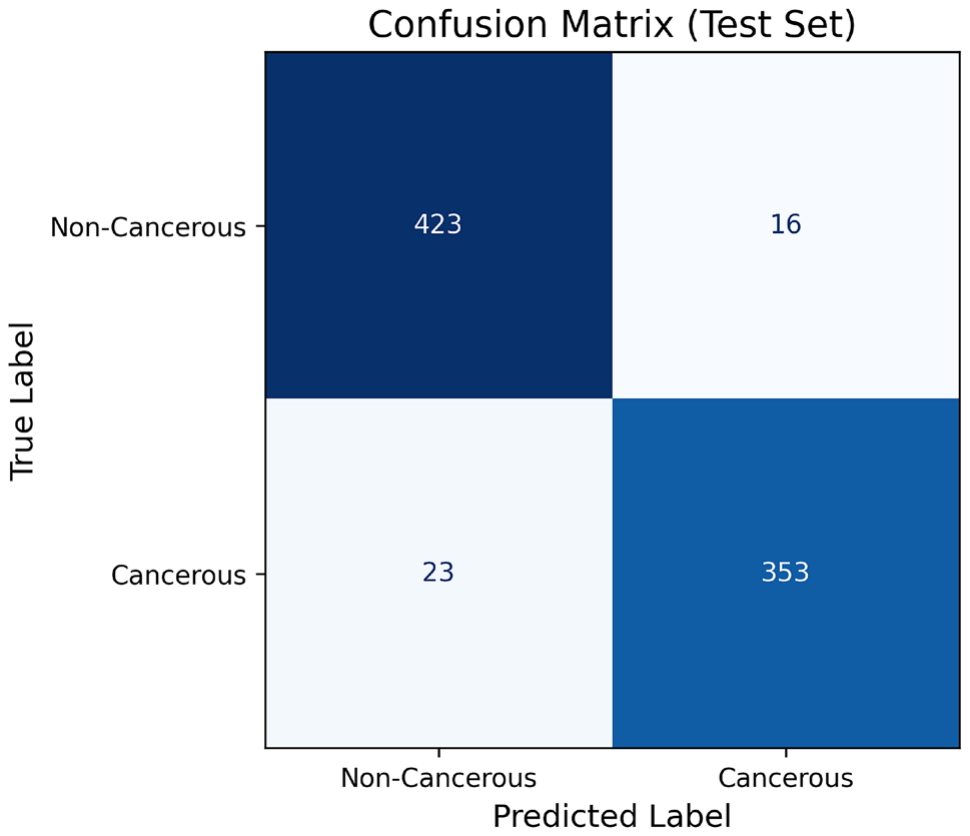
The confusion metric of the proposed hybrid model for lung cancer classification.

**Fig. 7:**
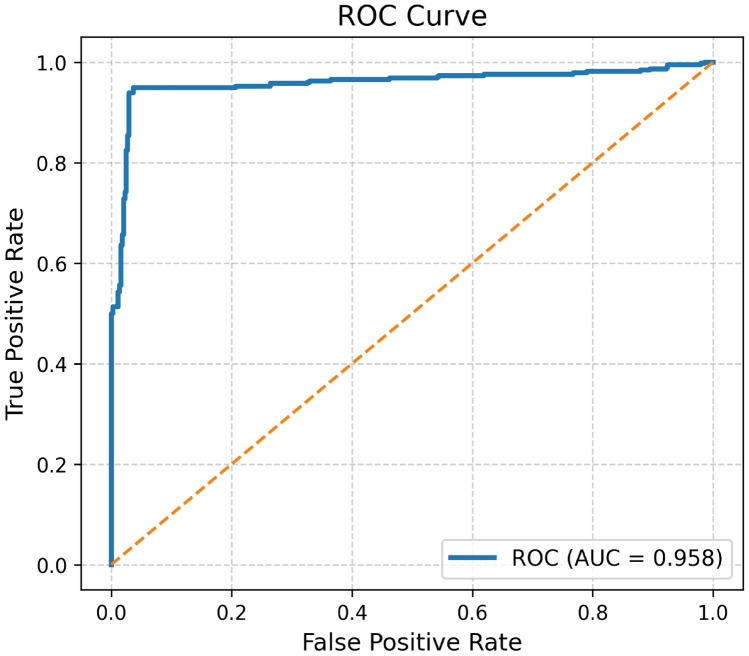
ROC curve of the proposed hybrid model for lung cancer classification.

**Fig. 8:**
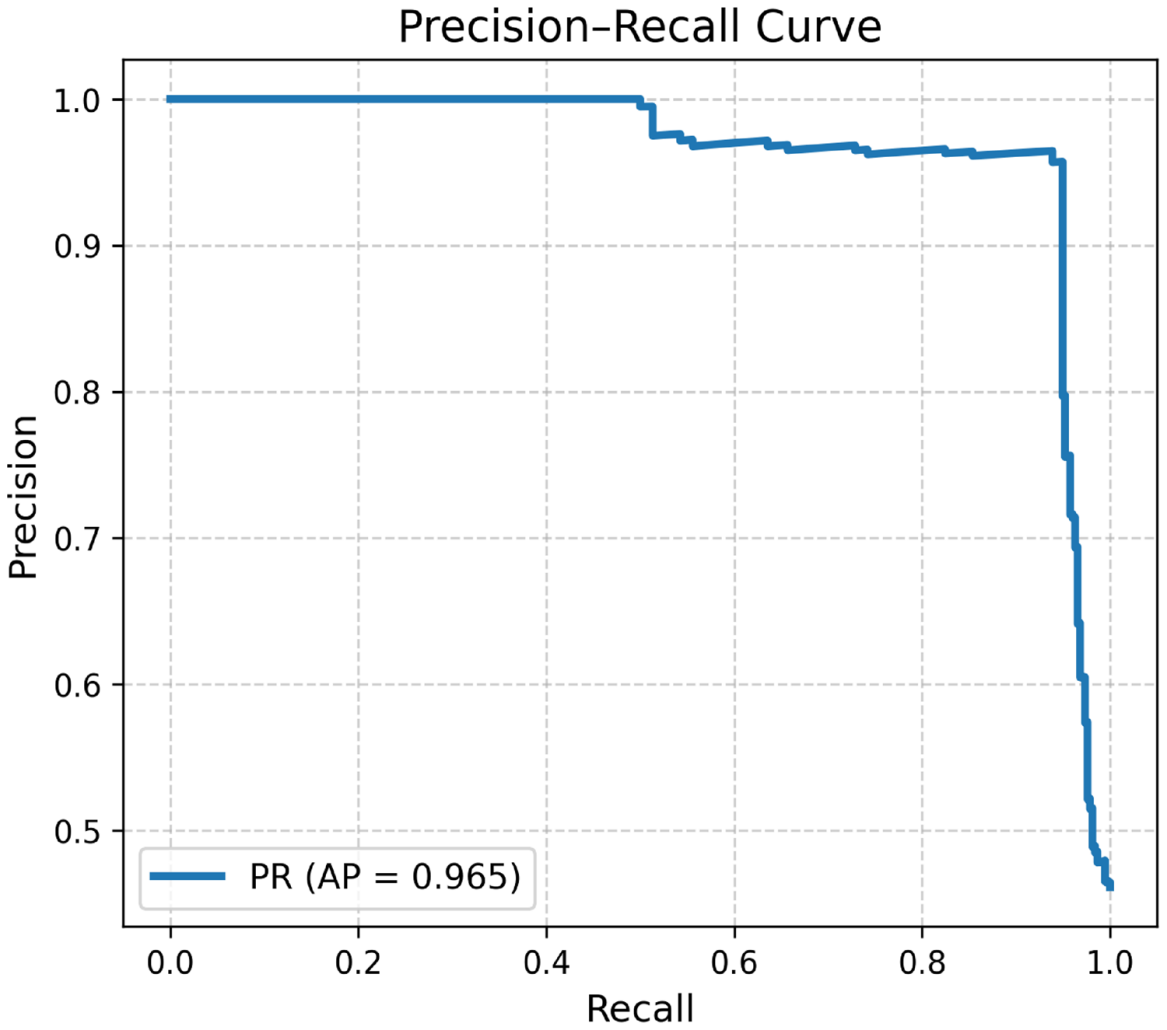
Precision–recall curve of the proposed hybrid model for lung cancer classification.

The Fig. 6 is used to display the confusion metric of the proposed model for lung cancer classification. The Fig. 7 is used to display the ROC curve of the proposed hybrid model for lung cancer classification. The main purpose of this curve is to display the performance of the model at different threshold values. The Fig. 8 is used to display the precision–recall curve of the proposed hybrid model for lung cancer classification. It is the trade off between precision and recall at different thresholds.

In addition, to estimate the effectiveness and importance of the proposed new hybrid deep learning model, a comparative analysis was performed. For a fair comparison, all baseline models were trained and tested under the same conditions as the proposed model. This analysis was performed for several state-of-the-art models which are commonly used to detect lung cancer with LDCT scan images. The valuation was done using standard metrics like Sensitivity, Specificity, Accuracy, AUC, F1-Score, and Dice Coefficient.

**Fig. 9:**
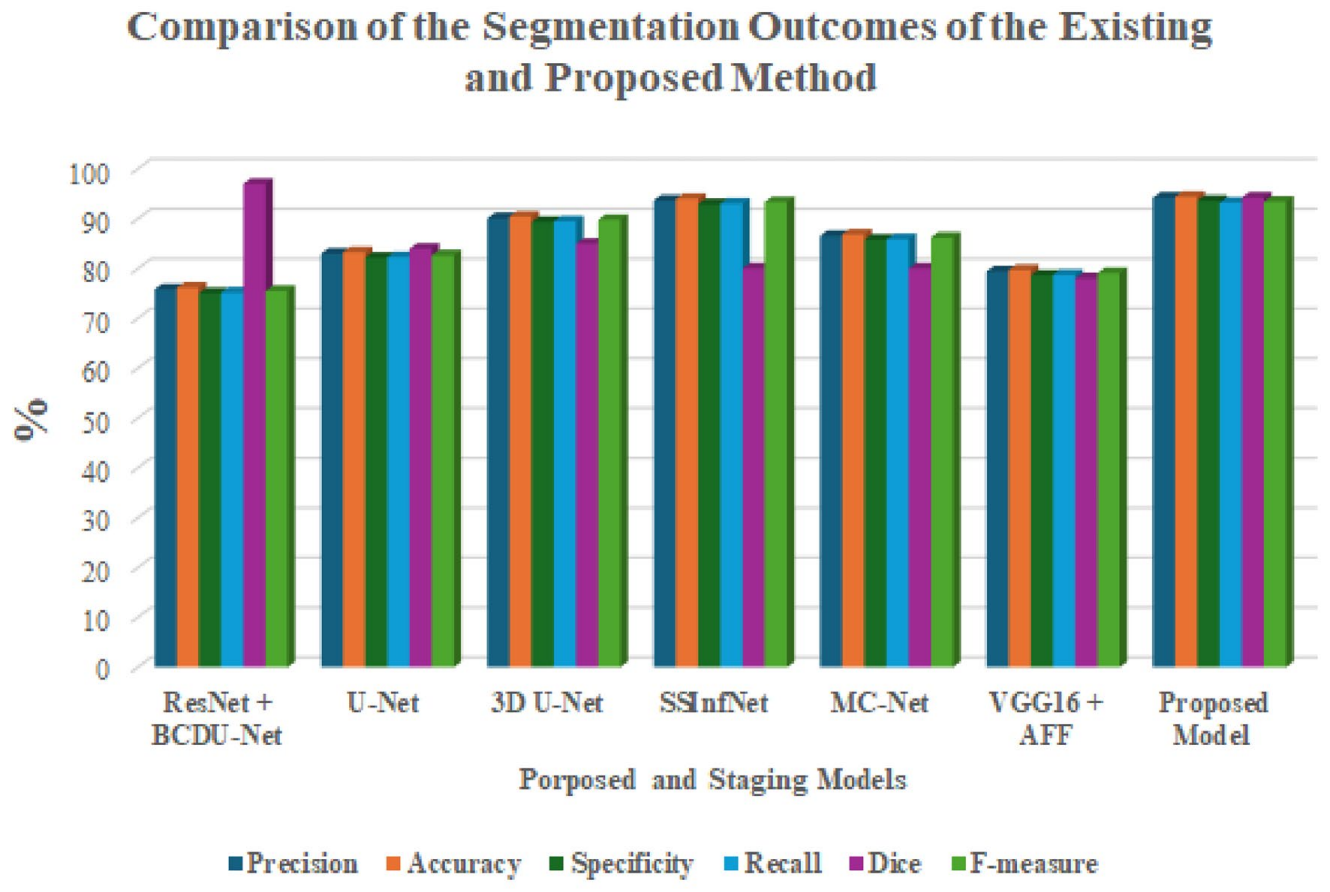
Comparison of the segmentation outcomes of the existing and proposed method.

**Fig. 10:**
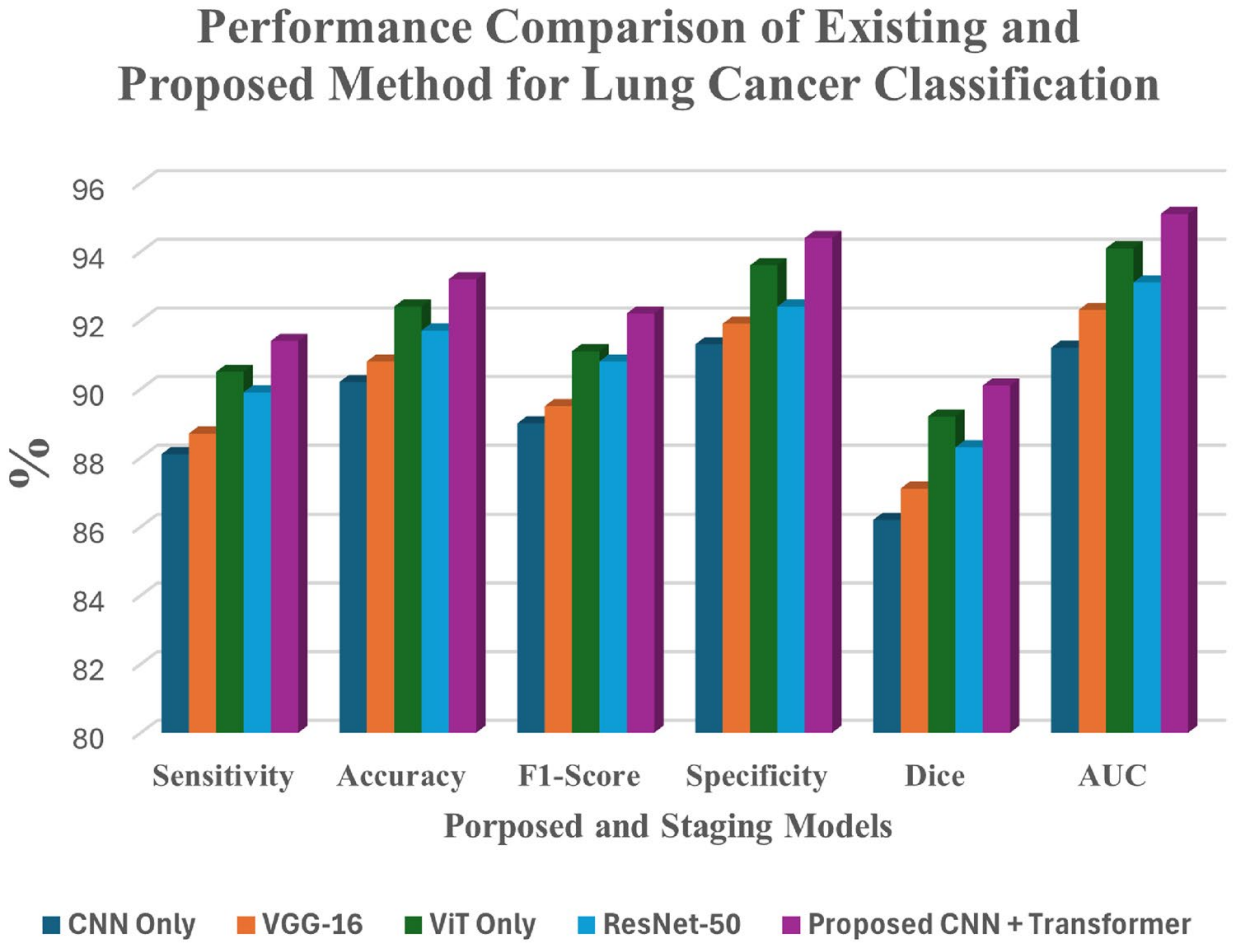
Performance comparison of existing and proposed method for lung cancer classification.

Table 6 is used to describe the experimental setup for the proposed hybrid model. Table 8 and Fig. 9 provides the comparison of the segmentation outcomes of the existing models with proposed method. Table 9 and Fig. 10 provides the comparative results of the classification outcomes to the proposed model with existing models.

### Qualitative results

**Table 6:** Experimental setup for proposed hybrid model execution.

Component	Specification
Operating system	Windows 10 Pro (x64-based, 64-bit)
Processor	Intel Core i7-9700K @ 3.60GHz
RAM	32GB DDR4 @ 3200MHz
GPU	NVIDIA GeForce GTX 1080 Ti, 12GB GDDR5X
Storage	256GB NVMe SSD
Programming language	Python 3.8

**Table 7:** Ablation study of the proposed hybrid pipeline.

Experiment	BM3D	YOLOv8 lung ROI	Classifier type	Accuracy (%)	Sensitivity (%)	AUC
E1	$\times$	$\times$	CNN only	89.4	87.2	0.91
E2	$\checkmark$	$\times$	CNN only	91.0	89.1	0.93
E3	$\times$	$\checkmark$	CNN only	92.2	90.5	0.94
E4	$\checkmark$	$\checkmark$	CNN only	93.1	91.7	0.95
E5	$\checkmark$	$\checkmark$	Transformer only	93.7	92.4	0.96
**E6(Proposed)**	$\checkmark$	$\checkmark$	**CNN+Transformer**	**95.1**	**93.8**	**0.97**

Significant values are in bold.

**Table 8:** Comparison of the segmentation outcomes of the existing and proposed method.

Model	Precision	Accuracy	Specificity	Recall	Dice(%)	F-measure
ResNet + BCDU-Net^23^	75.832	76.149	75.065	75.176	97	75.502
U-Net^24^	82.969	83.286	82.202	82.313	84	82.640
3D U-Net^25^	90.107	90.424	89.340	89.451	85	89.778
SSInfNet^26^	93.676	93.993	92.909	93.020	80	93.347
MC-Net^27^	86.538	86.855	85.771	85.882	80	86.209
VGG16 + AFF^28^	79.401	79.718	78.634	78.745	78	79.071
**Proposed hybrid model**	**94.234**	**94.436**	**93.621**	**93.231**	**94.231**	**93.418**

Significant values are in bold.

**Table 9:** Performance comparison of existing and proposed method for lung cancer classification.

Model	Sensitivity	Accuracy	F1-score	Specificity	Dice	AUC
CNN Only^29^	88.1%	90.2%	89.0%	91.3%	0.86	0.91
VGG-16^30^	88.7%	90.8%	89.5%	91.9%	0.87	0.92
ViT Only^31^	90.5%	92.4%	91.1%	93.6%	0.89	0.94
ResNet-50^32^	89.9%	91.7%	90.8%	92.4%	0.88	0.93
**Proposed hybrid model**	**93.8%**	**95.1%**	**94.4%**	**96.2%**	**0.92**	**0.97**

Significant values are in bold.

**Table 10:** Computational complexity comparison of baseline models and the proposed hybrid model.

Model	Input size	Trainable parameters (millions)	FLOPs (GFLOPs)
CNN Only	224$\times$224	3.2 M	1.8
VGG-16	224$\times$224	5.3 M	0.39
ViT-Base	224$\times$224	86.6 M	17.6
ResNet50	224$\times$224	25.6 M	4.1
Transformer encoder only	224$\times$224	2.1 M	1.2
**Proposed hybrid CNN +**	224$\times$224	**5.6 M**	**3**
**Transformer**			

Significant values are in bold.

### Baseline models for comparison

The following models were selected as baselines for the comparative study:

- CNN only: a traditional convolutional neural network without Transformer layers^29^.
- VGG-16: a standard CNN with deep feature extraction layers^30^.
- Vision transformer (ViT): a transformer-based model without convolutional stages^31^.
- ResNet-50: a deep residual network commonly used in medical image analysis^32^.

### Analysis

As evident from Table 9, the new proposed hybrid model outperforms the baseline models on all metrics of evaluation. The implementation of the Transformer Encoder into the model can capture global contextual information from the CNN model. This implementation complements the spatial feature extraction capabilities of the CNN module. In clinical applications where minimizing false negatives is essential, this technique leads to a marked improvement in AUC and sensitivity. Further, Table 10 is used to display the computational complexity of different baseline models with the proposed model using the same input size (224$\times$224). It is clear from table that ViT-Base Model is the most computationally expensive model with 86.6M parameters and 17.6 GFLOPs. The VGG-16 is the lightest model with only 0.39 GFLOPs. The proposed hybrid model achieves a good balance, requiring only 5.6M parameters and 3 GFLOPs. This will make it much more efficient than heavy transformer models while still maintaining moderate complexity compared to standard CNNs.

### Discussion and challenges

The proposed BM3D + YOLOv8 + hybrid CNN–Transformer framework has practical relevance for LDCT-based lung cancer screening, where early-stage malignancies are often subtle and radiologist workload is high. In clinical settings, false negatives are particularly critical, as missed malignant nodules may delay diagnosis and treatment. Therefore, the model can be configured to prioritize sensitivity through appropriate threshold tuning based on screening requirements. The pipeline can support LDCT screening workflows as a triage and decision-support tool, where scans predicted as high-risk may be prioritized for radiologist review. The YOLOv8 lung ROI segmentation improves robustness by removing irrelevant background regions and focusing the classifier on lung structures. While the suggested hybrid deep learning model provides very good results for early lung cancer detection from LDCT scan images, further there are many issues and limitations which are given below and need to be explored:

The model was trained and tested on publicly available datasets such as LIDC-IDRI. This dataset is well-annotated dataset. But real-life datasets which are captured from different scanner types, populations, and institutions, may or may not fully capture the diversity of clinical imaging. These datasets may require annotations as preprocessing steps. Generally, it is found that most of the publicly available dataset contains smaller proportion of malignant cases as compared to normal scans or benign cases. This kind of class imbalance may skew the model to the dominant class. This may cause false results. Coupling Transformer encoders with CNNs adds model complexity and computational cost. This will potentially constrain real-time deployment, particularly in low-resource or point-of-care environments.

While deep learning models achieve high accuracy, but these models work as black boxes. These models do not provide interpretability and explainability. This may limit clinical acceptance, as clinicians and radiologists require an understandable and logical reasoning behind the automated decisions. To remove noise and artifacts, accurate preprocessing and segmentation are very important. These preprocessing techniques are very important to provide better performance. Any errors or noise introduced during this step may affect the final output of the model. Generally, Deep learning-based hybrid models are trained on homogeneous or small datasets. These models are prone to overfitting. While regularization and augmentation strategies were employed to reduce overfitting risk. But generalization for uncategorized clinical data remains a challenge. This study mainly focuses on LDCT images. The future research should focus on additional data modalities like PET scans, clinical reports, and biomarkers. This will further improve robustness and diagnostic accuracy.

The next stage of study will focus on addressing these issues. Future research should focus on developing lightweight models and validating them on larger, multi-centre datasets. The future model should be multi-modal data fusion and deal with enhanced explainability strategies.

## Conclusion and future work

In this research, a new hybrid deep learning model that combines CNNs and Transformer Encoders was developed for early lung cancer detection using LDCT scan images. The model combines the best features of Transformers, which are good at modelling global contextual patterns, and CNNs, which are good at extracting local spatial patterns. Additionally, BM3D filtering as advanced preprocessing technique was applied to enhance model performance by reducing noise and improving image quality. The method was tested rigorously on the LIDC-IDRI dataset and compared with several state-of-the-art architectures. Performance results showed that hybrid architecture performed better than traditional models in accuracy, sensitivity, specificity, AUC, and Dice coefficient. This highlights its potential practicality as a robust and reliable approach for computer-aided lung cancer detection.

Despite its promising results, the study acknowledges many drawbacks, such as high processing costs, an unbalanced dataset, and model lack of interpretability. These points identify areas for future improvement. Future work will focus on various directions. First, escalating the model to include multi-modal data such as genetic biomarkers, clinical records, and PET imaging. This will help to improve diagnostic accuracy. Second, to enhance the transparency and foster clinical trust of the model it will deal with explainable AI (XAI) techniques. Real-world application will also need implementing the model in clinical workflows across various healthcare settings and tuning it for real-time inference. Finally, the model’s validation on larger and more heterogeneous datasets will increase the readiness and generalizability of the model for real-world applications.

## Data Availability

The LIDC-IDRI dataset used in this study is publicly available. The Code is available on request from Corresponding author (Gagan Thakral).
